# Time Spent Gaming, Device Type, Addiction Scores, and Well-being of Adolescent English Gamers in the 2021 OxWell Survey: Latent Profile Analysis

**DOI:** 10.2196/41480

**Published:** 2022-11-18

**Authors:** Simona Skripkauskaite, Mina Fazel, the OxWell Study Team

**Affiliations:** 1 Department of Experimental Psychology University of Oxford Oxford United Kingdom; 2 Department of Psychiatry University of Oxford Oxford United Kingdom

**Keywords:** gaming, adolescents, latent profile analysis, mobile phone, well-being, mental ill-health, mental health, digital technology

## Abstract

**Background:**

The shift in the last decades to screen-based and increasingly web-based gaming activity has raised concerns about its impact on the development of children and adolescents. Despite decades of research into gaming and related psychosocial effects, the question remains how best to identify what degree or context of gaming may be a cause for concern.

**Objective:**

This study aimed to classify adolescents into gamer profiles based on both gaming behaviors and well-being. Once we distinguished the different gamer profiles, we aimed to explore whether membership to a specific profile could be predicted based on a range of personal characteristics and experiences that could then help identify those at risk.

**Methods:**

We explored gaming and well-being in an adolescent school population (aged 12-18 years) in England as part of the 2021 OxWell student survey. Self-report measures of time spent playing games on computers or consoles, time spent playing games on mobile phones, the Game Addiction Scale, and the Warwick-Edinburgh Mental Well-being Scale were used to classify adolescent heavy gamers (playing games for at least 3.5 hours a day) using latent profile analysis. We used multinomial logistic regression analysis to predict the profile membership based on a range of personal characteristics and experiences.

**Results:**

In total, 12,725 participants answered the OxWell gaming questions. Almost one-third (3970/12,725, 31.2%) indicated that they play games for at least 3.5 hours a day. The correlation between time spent playing video games overall and well-being was not significant (*P*=.41). The latent profile analysis distinguished 6 profiles of adolescent heavy gamers: *adaptive computer gamers* (1747/3970, 44%); *casual computer gamers* (873/3970, 22%); *casual phone gamers* (595/3970, 15%); *unknown device gamers* (476/3970, 12%); *maladaptive computer gamers* (238/3970, 6%); and *maladaptive phone gamers* (79/3970, 2%). In comparison with *adaptive computer gamers*, *maladaptive phone gamers* were mostly female (odds ratio [OR] 0.08, 95% CI 0.03-0.21) and were more likely to have experienced abuse or neglect (OR 3.18, 95% CI 1.34-7.55). *Maladaptive computer gamers*, who reported gaming both on their mobile phones and on the computer, were mostly male and more likely to report anxiety (OR 2.25, 95% CI 1.23-4.12), aggressive behavior (OR 2.83, 95% CI 1.65-4.88), and web-based gambling (OR 2.18, 95% CI 1.24-3.81).

**Conclusions:**

A substantial number of adolescents are spending ≥3.5 hours gaming each day, with almost 1 in 10 (317/3970, 8%) reporting co-occurring gaming and well-being issues. Long hours gaming using mobile phones, particularly common in female gamers, may signal poorer functioning and indicate a need for additional support. Although increased time gaming might be changing how adolescents spend their free time and might thus have public health implications, it does not seem to relate to co-occurring well-being issues or mental ill-health for the majority of adolescent gamers.

## Introduction

### Background

Significant behavioral changes take place with every generation; these are often accompanied by concern in people working with these populations. The shift in the last decades to screen-based and increasingly web-based gaming activity has raised concerns in published commentaries and the popular press about how this might affect the developing child and adolescent [1]. Nevertheless, games have always been a hallmark of childhood and adolescence, and video gaming can be both a positive and a negative experience [2,3]. However, despite decades of research into gaming and related psychosocial effects, the question remains how best to identify what degree or context of gaming may be a cause for concern. In an environment of increasing mental health difficulties [4] as well as digital technology use [5-8], we decided to explore gaming and well-being profiles in an adolescent school population (aged 12-18 years) in England as part of the 2021 OxWell student survey, which was conducted during the COVID-19 pandemic.

Determining when gaming may be a sign of impaired functioning is complex because intensive video game use in itself does not necessarily equate to problematic gaming. Although traditionally studies on video game or digital media use have found negative associations with well-being [9-12], a growing body of recent evidence from large-scale studies shows that direct links between time spent engaging with digital technology and adolescent well-being or mental ill-health are either nonexistent or weak [13-17]. Many researchers argue instead that there may be a minority of gamers for whom gaming can become problematic and interfere with psychological and social functioning [8,18,19]. Despite the ongoing debate about the nature and existence of problematic gaming [20-23], a new diagnosis for gaming disorder is now included in the International Classification of Diseases, Eleventh Revision [24]. Gaming addiction measures may be able to capture problematic gaming via impaired self-regulation and a loss of control over gaming, said to affect approximately 2% to 9% of adolescent gamers [25]. Such problematic gaming has been repeatedly shown to correlate with multiple negative psychosocial correlates, including aggressive behaviors, depression, loneliness, poor sleep quality, and lower social competence [18,26]. Nevertheless, the links between the scores on the Game Addiction Scale (GAS) and time spent playing video games as well as negative correlates are also not linear and likely context dependent [27-29].

Focusing on average patterns of association, as is done in correlational studies, can mask the heterogeneity of the gamer population. Person-centered approaches such as latent class analysis offer an opportunity to explore such heterogeneity by identifying unobserved (latent) subgroups that are inferred from a set of observed variables [30]. Most of the previous studies attempting to classify adolescent gamers have approached gaming as a disorder and only devised subgroups based on their gaming addiction score per individual item [31,32]. One study [33] categorized adolescent gamers based on their weekly web-based gaming time in addition to compulsive internet use–scale scores and distinguished, among others, addicted and not-addicted heavy gamer classes. However, they did not find clear relationships between these classes and mental ill-health. One possible explanation for their findings is that mental ill-health does not necessarily capture all aspects of successful functioning and is not the same as poor well-being [34]. However, this also suggests that well-being should be directly accounted for when classifying adolescent gamers to better understand how gaming habits may differ among those with impaired functioning or those with an inability to control their gaming habits.

Many of the studies of gaming behaviors among adolescents focus on PC games or massive multiplayer online role-playing games and have not included mobile phone game use. They have found that both gaming and higher gaming addiction scores are more prevalent in male adolescents [35]. Nevertheless, gaming is increasing in popularity among girls aged 5 to 15 years [3], and smartphone use is more prevalent in girls and women [36]. Paik et al [37] have described patterns of gaming behaviors across different gaming devices in a Korean adult sample. Although male gamers reported predominantly playing computer games, and female gamers reported predominantly playing mobile phone games, those who played games evenly on both a computer and a mobile phone were evenly distributed across the genders. This group also had the highest prevalence of depression, anxiety, and internet gaming disorder. Given that smartphone gaming has seen a rise in recent years, with 58% of those aged between 16 and 24 years reporting playing games on their mobile phones in 2020-2021 compared with 47% in 2019 and 31% in 2012 [38], smartphone gaming is also likely to play a role in adolescent gaming patterns.

### Objectives

To best distinguish between those who engage in adaptive versus maladaptive gaming patterns, this study aimed to classify adolescent gamer profiles based not only on their gaming behaviors but also on their well-being. Specifically, we used a data-driven person-centered approach to explore whether latent gamer profiles can be determined based on how much time adolescents spend gaming on computers or consoles and mobile phones, their GAS scores, and their well-being. Once we distinguished the different gamer profiles, we aimed to explore whether their profile membership could then be predicted based on a range of personal characteristics and experiences that could help identify those at risk. These included sociodemographic information, specific gaming behaviors, school-related experiences and activities, family risk factors, and mental ill-health.

## Methods

### Study Design and Procedure

The OxWell student survey is a repeated cross-sectional survey of students, sampled from schools across 4 regions in England as described in the study protocol [39]. The OxWell survey collects data on a range of questions on mental ill-health and well-being, life experiences, and behaviors. It has 3 age-appropriate versions (divided into English school years 5 to 7, 8 to 11, and 12 to 13 and covering ages 9 to 18 years). The data analyzed here were collected from students in school years 8 to 13 in June and July 2021, a period during which schools were open, and most students had returned to in-person learning, but there were some classrooms affected by clusters of COVID-19 infection, causing whole classes to isolate. Participation in the OxWell survey was voluntary, and participants did not receive any monetary incentives to take part in the study.

### Ethics Approval

The study was approved by the research ethics committee of the University of Oxford (R62366).

### Participants

In total, 20,780 eligible students, based on predefined inclusion criteria [40], aged 12 to 18 years completed the OxWell survey in 2021. Of these 20,780 students, 8055 (38.76%) were excluded because of missing responses on gaming questions. To ensure survey completion during the designated school period (up to 45 minutes), the data on time spent gaming on a computer or console and a mobile phone, as well as from the GAS, were only collected from a subsample of participants. As previous research suggests that >4 hours of daily device-based engagement [41] or video gaming [42] is more likely to indicate impaired psychosocial functioning, only those participants who answered that they play games for at least 3.5 hours overall were asked these more targeted questions (“About how many hours a day do you usually play games on an electronic device [eg, computer, game console or phone]?”). Of the remaining 12,725 students, 8755 (68.8%) were excluded from further analysis because they were not playing for at least 3.5 hours and so were categorized as nongamers, resulting in a final sample of 3970 (31.2%) gamers (Table S1 in Multimedia Appendix 1).

### Measures

#### Classification Variables

##### Time Spent Gaming

Those participants who reported playing games on electronic devices for at least 3.5 hours a day were asked to provide more precise information on how many hours a day they usually spend playing games on a computer or games console (*computer gaming*) and their mobile phone (*phone gaming*). Participants were asked to respond using a slider scale ranging from *0 hours* to *4 hours or more*. The responses were recoded into 2 discrete 5-point scales (0 to 4) for computer gaming and phone gaming.

##### Gaming Addiction

Participants who reported playing games on electronic devices for at least 3.5 hours a day were also asked to self-report on the short version of the GAS [25]. The short scale asks participants about their experiences with games over the last 6 months and aligns with the main criteria of internet gaming disorder in the Diagnostic and Statistical Manual of Mental Disorders, Fifth Edition [43], and gaming disorder in the International Classification of Diseases, Eleventh Revision [44]. The items assess 7 addiction criteria: salience, tolerance, mood modification, relapse, withdrawal, conflict, and problems. All items are scored on a 5-point Likert scale ranging from 1 (*never*) to 5 (*very often*). These scores are averaged to represent a total GAS score. Generally, the GAS has been shown to have strong convergent and criterion validity and fair-to-excellent reliability [45].

##### Well-being

Adolescent self-reports on the Warwick-Edinburgh Mental Well-being Scale (WEMWBS) [46] were used to measure mental well-being. The WEMWBS comprises 14 positively phrased items that capture both feeling good and functioning well. Agreement with each item is indicated on a Likert scale ranging from 1 (*none of the time*) to 5 (*all the time*). Item scores are summed to produce a total score ranging from 14 to 70, with higher scores representing higher levels of mental well-being. The WEMWBS has been shown to be a psychometrically strong population measure of mental well-being and suitable for use with adolescent samples [47].

#### Predictor Variables

Participants reported on a number of personal characteristics and experiences that were examined as potential predictors of gamer profiles in this study. These included sociodemographic information such as age and gender as well as specific gaming behaviors such as playing video games before sleep (*late gaming*), experience of web-based gambling, or spending money on in-game purchases. Participants were also asked about school-related experiences and activities, including whether they felt a sense of belonging to the school community and how easy they found it to make and keep friends; experiences of school detention, aggressive behaviors, and bullying; and exercise frequency, as well as potential family risk factors, including whether they felt safe in the place they live, food poverty as a proxy for deprivation, and experiences of child abuse. Finally, a few different aspects of mental ill-health were examined, including anxiety and depression measured using the 25-item Revised Children’s Anxiety and Depression Scale [48], insomnia measured using the 2-item version of the Sleep Condition Indicator [49], loneliness based on the 3-item version of the UCLA Loneliness Scale [50,51], and lifetime self-harm [52,53]. Full details of the measures used as predictor variables in the study are provided in Table S2 in Multimedia Appendix 1 [48-51] and the preregistration for this analysis [40].

### Data Analysis

A latent profile analysis (LPA) using general mixture modeling was conducted in Mplus (Muthén & Muthén) [54] to determine latent profiles based on participants’ scores on 4 measures: computer gaming, phone gaming, GAS, and WEMWBS. LPA allows obtaining the probability that individuals belong to different groups, thus exposing hidden groups in the data [55]. Two 3-latent–profile models were initially fitted to determine whether profile covariance should be set to zero or constrained to be equal among profiles. A Satorra-Bentler scaled chi-square [56] test confirmed that the introduction of equality constraints significantly improved model fit (*χ*^2^_SB6_=701.2; *P*<.001). Therefore, models with 1 to 6 latent profiles that allowed the means but not variance or covariance to vary among profiles was fitted. All models used maximum likelihood estimation with robust SEs. To avoid the model identification at local maxima, each model used a set of 1000 random starting values, with 250 that yielded the highest log-likelihood to be used in the final optimizations, and 500 iterations.

Iterative evaluations of models comparing model fit indices were used to select the best-fitting model. The relative fit indices Bayesian information criterion and Vuong-Lo-Mendell-Rubin adjusted likelihood ratio [57,58] test were used to determine whether additional profiles in the LPA model improved the model fit.

In the second part of the analysis a multinomial logistic regression using *mlogit* package in R (version 4.1.3; The R Foundation for Statistical Computing) [59] was carried out to predict class membership using the categorical predictor variables. The individuals were assigned to their most likely profile using the posterior probability weights from the LPA to account for the assignment uncertainty. Next, their class membership was regressed onto the covariates (*gender*, *age*, *late gaming*, *tried web-based gambling*, *in-game purchases*, *school community*, *friendships*, *detention*, *aggression*, *bullying*, *exercise*, *sense of safety*, *food poverty*, *abuse*, *anxiety*, *depression*, *insomnia*, *loneliness*, and *self-harm*). Odds ratios (ORs) were used to determine the likelihood of association between the predictor variables and the profiles [60], and 95% CIs for the ORs were extracted to determine the significance of the association (ie, the 95% CIs should not cross the value of 1 to be reliable).

## Results

### Sample Characteristics and Spearman Correlation

In total, 12,725 participants answered the OxWell survey gaming questions, of whom 3970 (31.2%) *gamers* indicated that they play games on an electronic device for at least 3.5 hours a day, whereas 2779 (21.84%) reported not playing any games at all. The Spearman correlation between time spent playing video games overall and well-being was not significant when examined in the full sample (*r*_12,214_<–0.01; *P*=.98). However, in the sample of gamers (Table 1), well-being was positively correlated with the amount of time spent playing video games on a computer or console but negatively correlated with the amount of time spent playing video games on a mobile phone and GAS scores. Of the 3970 gamers, 1798 (45.29%) had missing information on ≥1 predictor variable. To use the maximum available data, the full sample of gamers (n=3970) was included in the LPA classification, and the data from the adolescent gamers without missing predictor information (2172/3970, 54.71%) were used for the multinomial logistic regression (a comparison of excluded and included participants is presented in Tables S3 and S4 in Multimedia Appendix 1). Participant characteristics per analytical sample are described in Table 2.

**Table 1:** Spearman correlation matrix for classification variables.

Variable		Computer gaming	Phone gaming	GAS^a^	WEMWBS^b^
**Computer gaming**					
	*r*	1	–0.03	0.37	0.12
	*P* value	—^c^	.04	<.001	<.001
**Phone gaming**					
	*r*	–0.03	1	0.17	–0.09
	*P* value	.04	—	<.001	<.001
**GAS**					
	*r*	0.37	0.17	1	–0.29
	*P* value	<.001	<.001	—	<.001
**WEMWBS**					
	*r*	0.12	–0.09	–0.29	1
	*P* value	<.001	<.001	<.001	—

^a^GAS: Game Addiction Scale.

^b^WEMWBS: Warwick-Edinburgh Mental Well-being Scale.

^c^Not applicable.

**Table 2:** Sample characteristics per categorical predictor variable for the classification sample (N=3970) and the prediction subsample (n=2172).

Characteristic			Classification sample, n (%)		Prediction subsample, n (%)
**Age (years)**					
	17 to 18	206 (5.2)		134 (6.2)	
	12 to 16	3764 (94.8)		2038 (93.8)	
**Gender**					
	Boy	2246 (56.6)		1416 (65.2)	
	Girl	1437 (36.2)		756 (34.8)	
	Other or prefer not to answer	287 (7.2)		0 (0)	
**Late gaming**					
	At least sometimes	3498 (88.1)		1961 (90.3)	
	Rarely	390 (9.8)		211 (9.7)	
	Missing	82 (2)		0 (0)	
**Tried web-based gambling**					
	Yes	425 (10.7)		234 (10.8)	
	No	3239 (81.6)		1938 (89.2)	
	Missing	306 (7.7)		0 (0)	
**In-game purchases**					
	Yes	3123 (78.7)		1794 (82.6)	
	No	718 (18.1)		378 (17.4)	
	Missing	129 (3.2)		0 (0)	
**School community**					
	Yes	674 (17)		456 (21)	
	No	3040 (76.6)		1716 (79)	
	Missing	256 (6.4)		0 (0)	
**Friendships**					
	Difficult	1782 (44.9)		953 (43.9)	
	Easy	1964 (49.5)		1219 (56.1)	
	Missing	224 (5.6)		0 (0)	
**Detention**					
	Several times	767 (19.3)		382 (17.6)	
	Once or twice	3112 (78.4)		1790 (82.4)	
	Missing	91 (2)		0 (0)	
**Aggression**					
	Yes	517 (13)		235 (10.8)	
	No	3316 (83.5)		1937 (89.2)	
	Missing	137 (3.5)		0 (0)	
**Bullying**					
	Bullied	303 (7.6)		135 (6.2)	
	Not bullied	3619 (91.2)		2037 (93.8)	
	Missing	48 (1.2)		0 (0)	
**Exercise (hours per day)**					
	>1	3270 (82.4)		1919 (88.4)	
	≤1	501 (12.6)		253 (11.6)	
	Missing	199 (5)		0 (0)	
**Sense of safety**					
	Unsafe	471 (11.9)		202 (9.3)	
	Safe	3422 (86.2)		1970 (90.7)	
	Missing	77 (2)		0 (0)	
**Food poverty**					
	Yes	683 (17.2)		323 (14.9)	
	No	3234 (81.5)		1849 (85.1)	
	Missing	53 (1)		0 (0)	
**Abuse**					
	Yes	929 (23.4)		457 (21)	
	No	3041 (76.6)		1715 (79)	
**Anxiety**					
	Above threshold	561 (14.1)		299 (13.8)	
	Below threshold	3077 (77.5)		1873 (86.2)	
	Missing	332 (8.4)		0 (0)	
**Depression**					
	Above threshold	743 (18.7)		383 (17.6)	
	Below threshold	2899 (73)		1789 (82.4)	
	Missing	328 (8.3)		0 (0)	
**Insomnia**					
	Yes	561 (14.1)		253 (11.6)	
	No	3346 (84.3)		1919 (88.4)	
	Missing	63 (2)		0 (0)	
**Loneliness**					
	Lonely	1720 (43.3)		834 (38.4)	
	Not lonely	2172 (54.7)		1338 (61.6)	
	Missing	78 (2)		0 (0)	
**Self-harm**					
	Yes	738 (18.6)		452 (20.8)	
	No	2348 (59.1)		1720 (79.2)	
	Missing	884 (22.3)		0 (0)	

After fitting models with 2 to 6 latent classes (Table S5 in Multimedia Appendix 1), the 6-class model yielded the best fit. The best model fit was based on the drop in the Bayesian information criterion and Vuong-Lo-Mendell-Rubin adjusted likelihood ratio comparison, and it was acceptable based on additional diagnostic criteria such as entropy index and smallest class size.

### Gamer Profiles

**Figure 1:**
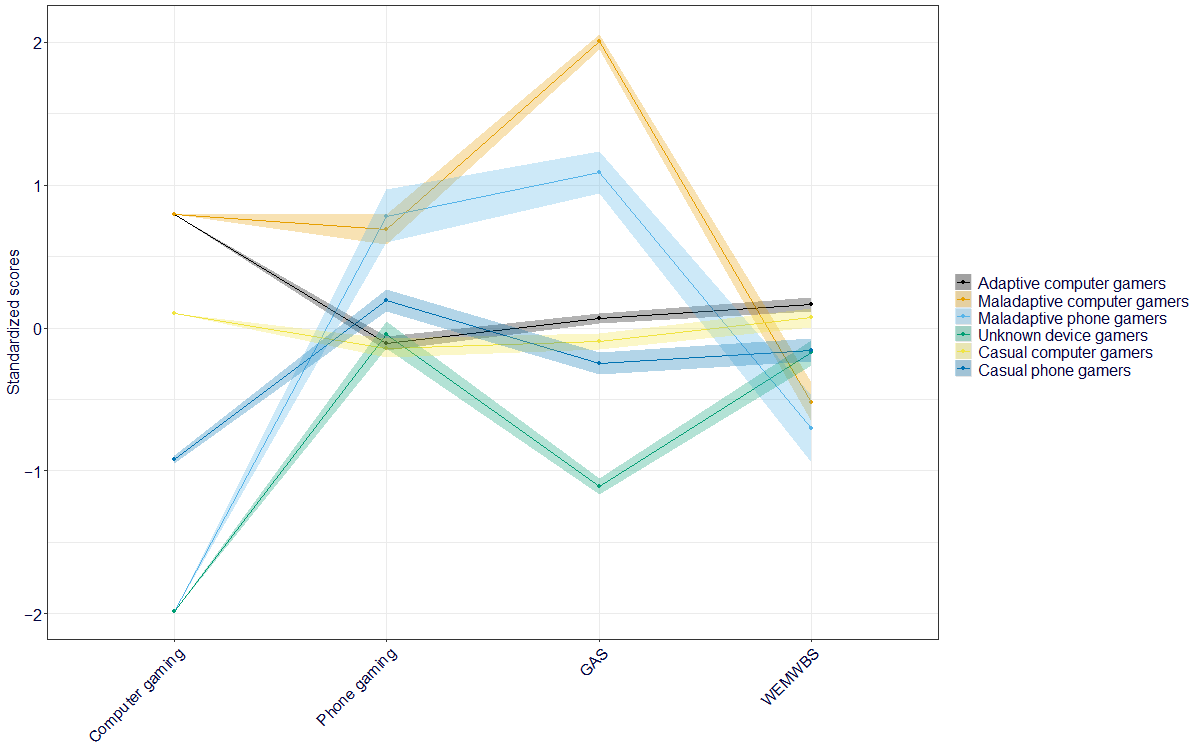
Estimated latent profiles for adolescent gamers. The y-axis represents scaled and centered values for each classification variable. The shaded area represents 95% CIs. GAS: Gaming Addiction Scale; WEMWBS: Warwick-Edinburgh Mental Well-being Scale.

From this model, 6 distinct gamer profiles emerged (Figure 1; Table 3). Half (1973/3970, 49.7%) of the participants fell into 2 profiles characterized by the maximum amount of computer gaming (≥4 hours). Specifically, 43.53% (1728/3970) of our sample were most likely to be in the *adaptive computer gamers* group characterized by high scores on computer gaming, relatively low scores on phone gaming, medium GAS scores, and the highest well-being, whereas 6.17% (245/3970) of the participants with high scores on computer gaming were characterized by longer hours playing games on their mobile phone, the highest GAS scores, and lower well-being and thus were deemed to fall into the *maladaptive computer gamers* group.

**Table 3:** Means and SDs of classification variables for gamer (n=3970) profiles and nongamers (n=8755).^a,b,c^

	Adaptive computer gamers	Maladaptive computer gamers	Maladaptive phone gamers	Unknown device gamers	Casual computer gamers	Casual phone gamers	Nongamers
Computer gaming, mean (SD)	4.00^d^ (0.00)	4.00^d^ (0.00)	0.00^e^ (0.00)	0.00^e^ (0.00)	3.00 (0.00)	1.53 (0.50)	N/A^f^
Phone gaming, mean (SD)	1.46^g,h^ (1.21)	2.42^i^ (0.99)	2.54^i^ (0.93)	1.53^g,j^ (1.30)	1.42^h,j^ (1.17)	1.82 (1.10)	N/A
GAS^k^, mean (SD)	2.60 (0.70)	4.43 (0.40)	3.56 (0.58)	1.49 (0.55)	2.45 (0.78)	2.31 (0.86)	N/A
WEMWBS^l^, mean (SD)	45.70^m^ (11.30)	37.80^n^ (12.60)	35.60^n^ (11.10)	41.70^m,o^ (10.90)	44.60^p^ (11.30)	41.90^o^ (11.10)	44.50^p^ (10.70)

^a^Nongamers include participants who reported playing games for <3.5 hours a day.

^b^The information on missing data regarding classification variables per profile is presented in Table S6 in Multimedia Appendix 1.

^c^Means that do not share the same superscript letters are significantly different (*P*<.001).

^d^*P*=.99.

^e^*P*=.99.

^f^N/A: not applicable.

^g^*P*=.28.

^h^*P*=.46.

^i^*P*=.48.

^j^*P*=.13.

^k^GAS: Game Addiction Scale.

^l^WEMWBS: Warwick-Edinburgh Mental Well-being Scale.

^m^*P*=.02.

^n^*P*=.16.

^o^*P*=.81.

^p^*P*=.89.

Two further profiles encompassed a relatively small number of participants who only engaged with phone, rather than computer or console, gaming. The smallest profile of *maladaptive phone gamers* characterized 1.74% (69/3970) of the participants, who did not spend any time playing computer games but spent the longest time playing on mobile phones. They were also characterized by high GAS scores and the lowest average well-being in the sample. The other group that reported not playing computer games included 12.04% (478/3970) of the participants, who engaged in some gaming on their mobile phones but had the lowest GAS scores and reported medium well-being. As all participants in the sample previously reported playing games for at least 3.5 hours a day, this group will be referred to as *unknown device gamers*.

The final 2 profiles encompassed more than a third (1450/3970, 36.52%) of the participants, who played some computer games but not as much or as little as the other classes. Most (873/3970, 22%) were characterized by relatively high computer gaming, relatively low phone gaming, GAS scores just below average, and high well-being. This group was named *casual computer gamers*. The rest (577/3970, 14.53%) were defined by relatively low computer gaming scores, medium phone gaming scores, below-average GAS scores, and medium well-being scores and were thus referred to as *casual phone gamers*.

**Figure 2:**
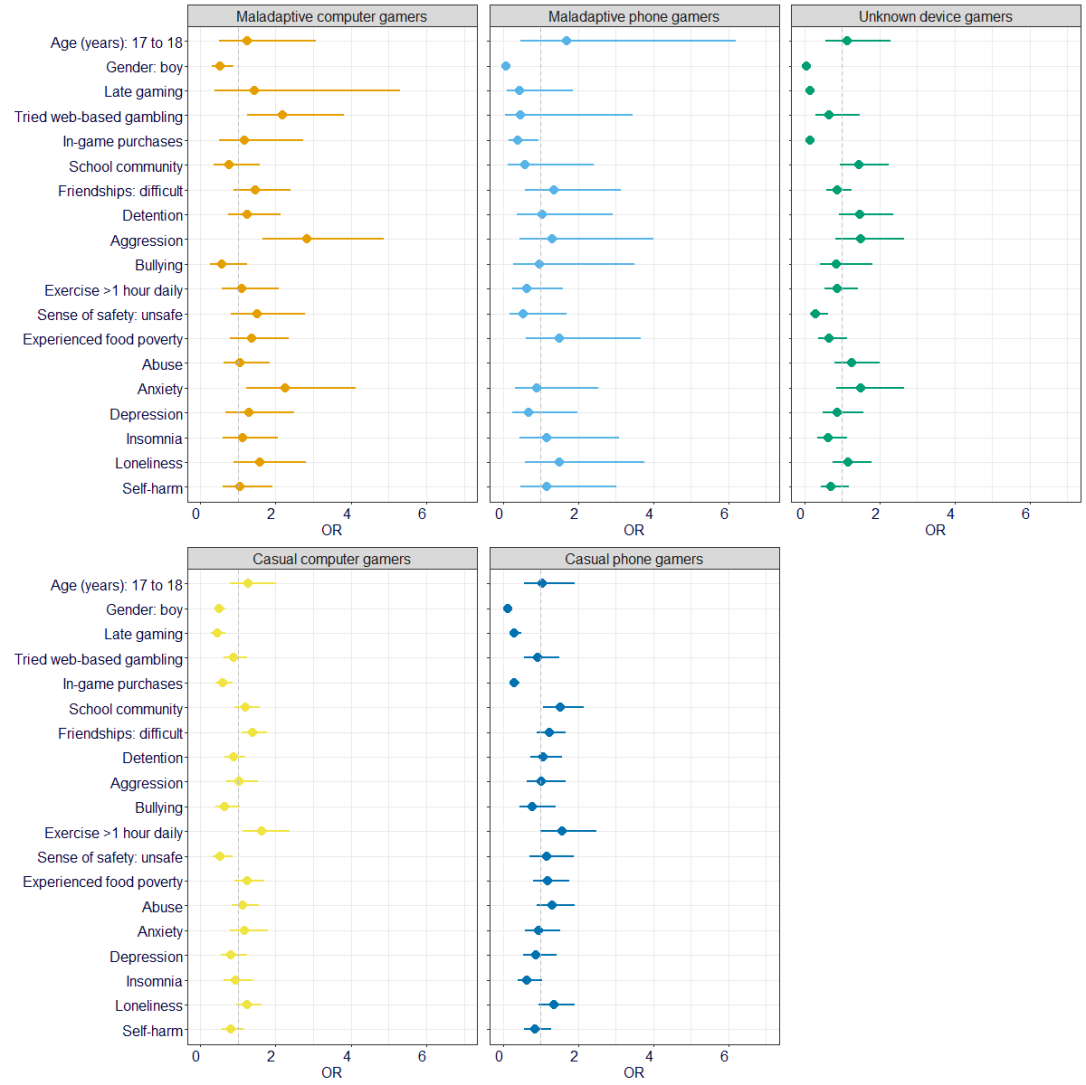
Relative odds ratios (ORs) comparing the likelihood of gaming profiles per hypothesized predictor variable (reference group: adaptive computer gamers). Error bars represent 95% CIs for the ORs. OR and 95% CI >1 (to the right of the dotted line) indicate an increased likelihood of belonging to one of these gamer groups compared with adaptive computer gamers, whereas OR and 95% CI <1 (to the left of the dotted line) indicate a decreased likelihood of belonging to one of these gamer groups compared with adaptive computer gamers.

Multinomial logistic regression indicated that the likelihood of being categorized into different gamer profiles could be based on some of the hypothesized predictor variables (Figure 2). For instance, participants in the *maladaptive computer gamers* group, in comparison with the *adaptive computer gamers* group, were less likely to be male (OR 0.51, 95% CI 0.30-0.88) and more likely to have reported anxiety symptoms above the clinical threshold (OR 2.25, 95% CI 1.23-4.12), to have said that they are often aggressive or violent (OR 2.83, 95% CI 1.65-4.88), or to have previously engaged in web-based gambling (OR 2.18, 95% CI 1.24-3.81). *Maladaptive phone gamers*, in comparison with the *adaptive computer gamers*, were even less likely to be male (OR 0.08, 95% CI 0.03-0.21) and less likely to report spending money on in-game purchases (OR 0.40, 95% CI 0.17-0.95) but were more likely to have experienced child abuse, neglect, or domestic violence (OR 3.18, 95% CI 1.34-7.55). Both *casual computer gamers* and *casual phone gamers* were less likely than *adaptive computer gamers* to be male (OR 0.50, 95% CI 0.38-0.67 and OR 0.14, 95% CI 0.10-0.20, respectively), to engage in late night gaming during the hour before sleep (OR 0.45, 95% CI 0.30-0.67 and OR 0.31, 95% CI 0.19-0.50, respectively), or to report spending money on in-game purchases (OR 0.60, 95% CI 0.42-0.86 and OR 0.31, 95% CI 0.21-0.45, respectively). Nevertheless, *casual computer gamers* were also less likely than *adaptive computer gamers* to express feeling unsafe in the place they live (OR 0.53, 95% CI 0.33-0.85) and more likely to say that they find it difficult to make friends (OR 1.39, 95% CI 1.09-1.76) or engage in >1 hour of daily exercise (OR 1.63, 95% CI 1.12-2.37). By contrast, *casual phone gamers* were more likely than *adaptive computer gamers* to state that they identify with their school community (OR 1.52, 95% CI 1.07-2.15). *Unknown device gamers* were least likely to be male (OR 0.04, 95% CI 0.03-0.06), to engage in late night gaming during the hour before sleep (OR 0.14, 95% CI 0.09-0.24), to report spending money on in-game purchases (OR 0.14, 95% CI 0.09-0.21), or to express feeling unsafe in the place they live (OR 0.30, 95% CI 0.14-0.62) compared with the *adaptive computer gamers*. Full characteristics of the 6 profiles are presented in Tables S7 and S8 in Multimedia Appendix 1. An exploratory analysis using the excluded nongamers as a reference category in the multinomial logistic regression is also included in Multimedia Appendix 1 (refer to Supplementary Analysis: Gamer Profiles in Comparison With Nongamers [Figures S1 and S2; Table S9]).

## Discussion

### Principal Findings

In this large school survey of the health and well-being of English students, almost one-third (3970/12,725, 31.2%) of the students who answered the questions on time spent on electronic devices said that they were gaming for at least 3.5 hours per day, whereas a fifth (2779/12,725, 21.84%) reported not engaging in any gaming. By examining time spent gaming per device type, GAS scores, and a well-being measure, 6 different gamer profiles emerged among those who were gaming the longest each day. The majority (1728/3970, 43.53%) of the students gaming for at least 3.5 hours fell into adaptive gaming categories with the highest well-being scores. Almost a tenth (314/3970, 8%) of the gamers exhibited maladaptive gaming patterns with the lowest well-being scores. Specifically, *maladaptive phone gamers* were a small group who were mostly female and were more likely to have experienced abuse or neglect. *Maladaptive computer gamers*, who reported gaming on their mobile phones in addition to computer gaming, were mostly male and more likely to report anxiety, aggressive behavior, and engagement in web-based gambling. Generally, those involved in predominantly computer gaming were mostly male, and those involved in predominantly phone gaming were mostly female.

### Comparison With Prior Work

Our findings support previous research showing that the amount of time spent playing video games does not necessarily indicate problematic gaming behavior [16,27,28]. Nearly half (1728/3970, 43.5%) of the gamers in this study engaged in ≥4 hours of computer gaming a day but reported high well-being. Overall, 8% (314/3970) of the adolescent gamers, corresponding to 2.47% (314/12,725) of the full sample, fell into the maladaptive gamer categories, which is also in line with previous estimates [25]. The *maladaptive computer gamers* group was most similar to problematic gamers identified in previous studies [18,26]. Specifically, this group not only spent large amounts of time playing video games daily but also reported low well-being and high GAS scores and were most likely to report aggressive behaviors and anxiety.

These findings expand on previous knowledge by showing that long hours of mobile phone, rather than computer or console, gaming may signal poor functioning. Two of the gamer groups that reported the highest phone gaming in this study also showed the highest GAS and lowest well-being scores. Paik et al [37] have previously found that adults who reported playing games on both their computers and mobile phones, rather than only on their mobile phones, were most likely to score higher on an internet gaming disorder scale and have higher depression and anxiety. Differently from their findings, we identified 2 maladaptive gamer groups that differed on their engagement with computer games, but both were characterized by playing games on their mobile phones for approximately 2.5 hours per day. Given the technological advances and wide availability of smartphones, with 93% of those aged 12 to 15 years in the United Kingdom owning a mobile phone [3], it seems realistic that those with the highest GAS scores would use these portable devices to meet their gaming needs.

In line with previous studies examining phone gaming [37] or smartphone use more generally [36], those engaged in predominantly phone gaming were more likely to be female than those engaged in predominantly computer gaming. Previous reviews highlight how female gamers experience a unique set of obstacles when engaging in video games, such as web-based harassment, hypersexualized female avatars, or aggressive gameplay [61]. It is plausible that gamers in this study who were female were also more likely to have had negative experiences during gameplay that, in turn, either motivated them to engage in phone gaming instead or had an impact on their well-being.

Our findings suggest that long hours spent gaming may be more typical in male adolescents but more likely to indicate problems in well-being for some female adolescents. The *maladaptive phone gamers* were mostly female, whereas the *maladaptive computer gamers* were mostly male. However, although nearly twice as many male gamers than female gamers were categorized into the *maladaptive computer gamers* group, they were still *more likely* to be female than the *adaptive computer gamers*. Female gamers were proportionally least likely to be assigned to the *adaptive computer gamers* group. Instead, they were proportionally most likely to fall into the *unknown device gamers* group that had the lowest GAS score on average but lower well-being than the *adaptive computer gamers* group. This is in line with previous research that found that female adolescents are particularly at risk for mental ill-health and lower well-being [62]. However, it is worth noting that those with previous experience of emotional abuse, neglect, or domestic violence were the most likely to fall into the *maladaptive phone gamers* group. Thus, it is also possible that female gamers who struggle with lower well-being because of previous traumatic experiences may seek out gaming, especially phone gaming, as a coping mechanism. This is partially in line with research showing that extrinsic or escapist motives, rather than playing for fun, are more likely to relate to negative gaming consequences [16,63,64].

A few other personal characteristics and experiences explored in this study predicted the membership of different gamer profiles, revealing a distinction between adaptive heavy gamers and more moderate gaming classes. For instance, *casual computer gamers* were having more difficulty making and keeping friends than adaptive gamers, but they were more likely to exercise. *Casual phone gamers* were most likely to identify with the school community, whereas *unknown device gamers* and *casual computer gamers* were more likely to feel safe at home compared with the adaptive gamers. This pattern of findings partially contradicts the displacement hypothesis [65], which would suggest that replacement of alternative activities such as socializing or exercising with gaming would be associated with lower, rather than higher, well-being. Instead, these findings suggest that gaming may be a potential coping strategy also used by those in, for example, unsafe environments, albeit with different associations for well-being than among those with previous experience of abuse who mostly fell in the *maladaptive phone gamers* group. Taken together, these findings support the theory of compensatory use outlined in the context of internet addiction, according to which negative life situations can give rise to a motivation to go on the web to alleviate negative feelings, the success of which may depend on the level of unmet needs [66]. However, the cross-sectional nature of this study limits our ability to make observations about the direction of effects. Future longitudinal research could disentangle these potential mediation patterns.

Our findings further suggest that some of the gaming-related behaviors that have been previously suggested to indicate risk behaviors for problematic gaming [18,26] may just be part and parcel of heavy daily gaming rather than specific to problematic gaming. For instance, making in-game purchases, although less common in the other groups, seemed to be a common characteristic among those playing extensive computer games and did not distinguish between adaptive and maladaptive gamers. Late night gaming was, not surprisingly, less common among those who engaged in less gaming overall but again did not distinguish between adaptive and maladaptive gamers. Nevertheless, experiences of web-based gambling did distinguish between *adaptive computer gamers* and *maladaptive computer gamers* in line with previous observed risks between gaming addiction and gambling [67].

### Practical Implications

Our findings suggest that certain groups of gamers are at greater risk for co-occurring gaming and well-being issues and may require support in dealing with behavioral difficulties and mental ill-health. This study extends previous research by showing that large amounts of time spent gaming on mobile phones, particularly common in female gamers, may signal poorer functioning, including aggressive behaviors and anxiety as well as experiences of abuse, neglect, or domestic violence. Although further longitudinal and experimental research is needed to understand the causal mechanisms behind this association, our findings highlight a potential avenue for mental health interventions with psychoeducational and therapeutic video (especially mobile phone) games as an opportunity to reach many adolescents struggling with mental ill-health. Indeed, as almost one-third (3970/12,725, 31.2%) of our sample reported playing video games for at least 3.5 hours a day, so did many of those with mental ill-health report heavy gaming (Table 2). This means that a substantial proportion of gamers across all groups, albeit especially in the maladaptive groups, could benefit from interventions for their reported anxiety, depression, insomnia, and self-harm. Certain video games have already been shown to help with symptoms of anxiety and depression [68], as well as be as effective as cognitive behavioral therapy [69] and more effective than second-line medication [70]. Rather than targeting time spent playing video games, using video gaming as a tool presents an opportunity for more affordable and less stigmatizing mental health interventions for adolescent populations and worthy of further investigation.

### Limitations and Future Directions

Findings from the study should be considered within its limitations. First, this study uses a cross-sectional design, which curbs our ability to ascertain directionality of the effects; for example, although we found that some (314/3970, 8%) of the adolescents who play video games for at least 3.5 hours also report high GAS scores and low well-being, we are unable to determine whether their well-being is a cause or a consequence of their gaming habits or entirely unrelated. We are also unable to determine what the longer-term effects of heavy gaming may be. Second, although the OxWell student survey is representative of children and adolescents aged 8 to 18 years attending schools or further education colleges in participating counties in England, only a proportion (12,725/20,780, 61.24%) of the full sample was included in this study. A large proportion (8055/20,780, 38.76%) of the participants had to be excluded because they did not answer the question on their gaming habits; these questions were placed toward the end of the survey, and therefore many students might not have been allocated sufficient time to complete all the questions (45 minutes). As only those who played video games for at least 3.5 hours a day were asked further questions on their gaming habits, those who reported playing video games for <3.5 hours were excluded from the main analyses. Therefore, it remains unclear how the gamer profiles or their correlates observed in this study generalize or compare with the gaming patterns of the adolescents reporting spending some, but not as much, time playing video games (5976/12,725, 46.96%). Further studies examining longitudinal patterns in gaming behaviors in adolescent populations will better elucidate how those with poorer well-being or problematic motivation differ in their video game habits. More in-depth clinical assessments could also provide further information on potential well-being and mental health effects not captured in this study.

Moreover, the timing of the data collection could also influence the findings observed. Although the data were collected during the school term, it is plausible that gaming behaviors observed would have been different if measured in autumn or winter; for example, in summer adolescents may be spending more time gaming because of longer daytime hours or less time gaming because they are spending more time outdoors. Similarly, adolescent well-being and mental health scores could also have been seasonally affected [71]. Furthermore, the data analyzed in this study were collected in the context of the COVID-19 pandemic. Both mental ill-health [4] and gaming [5-7] have been reported to have increased in children and adolescents during the pandemic. It is thus possible that our findings represent a time when gaming was used by adolescents more commonly than usual. However, research shows that mental ill-health symptoms were worse in children and adolescents during periods of higher COVID-19–related restrictions [72], and these data were collected in a period (June and July 2021) when restrictions were relatively low, with most students having returned to in-person learning. Nevertheless, the COVID-19 pandemic is likely to have long-term impacts on child and adolescent mental ill-health as well as their engagement with digital technology, potentially explaining inconsistencies between these findings and some of the previous research.

Finally, the screen-based behaviors of the population are rapidly changing, especially in the arena of gaming. The options available at any one time can be dramatically different from one period of time to another; therefore, many of the previous studies and questionnaires developed do not consider the latest innovations in the field, popularity of specific games, and patterns of behavior. In the 2021 OxWell student survey, questions asked students about their own mobile phone use but not about use of mobile phones belonging to their parents or another family member, which may explain the existence of the *unknown device gamers* group. The students were also not asked other gaming-related questions that might have further enhanced our knowledge, such as which games they were playing, the variety of their choice of games, and more specific patterns of use, including whether they played with their friends, with other individuals in web-based gaming communities, or alone. The developments in game variety, device accessibility, and tailored incentives show no signs of abating and are likely to draw more adolescents into gaming, warranting further study.

### Conclusions

This is one of the largest studies of adolescent gaming and well-being conducted in England. A substantial number of school-age children are spending at least 3.5 hours gaming each day. Nevertheless, the majority of young people spending much of their time gaming seem to be experiencing few negative effects with regard to their well-being, with <1 in 10 (317/3970, 8%) showing potentially maladaptive patterns of behavior. Our findings highlight how female gamers and those using their mobile phones are potentially at greater risk for co-occurring gaming and well-being issues and are important groups to better understand in order to support them if their difficulties become significant. Although increased time gaming might be changing how adolescents spend their free time and, thus, have public health implications, it does not seem to, at least cross-sectionally, relate to co-occurring well-being issues or mental ill-health for the majority of adolescent gamers.

## Appendix

Multimedia Appendix 1 Supplementary tables and analyses.

## Abbreviations

GAS: Game Addiction Scale
LPA: latent profile analysis
OR: odds ratio
WEMWBS: Warwick-Edinburgh Mental Well-being Scale

## Data Availability

All authors had full access to all the data in the study and accept responsibility for submitting the paper for publication. Fully deidentified extracts of the data can be provided to academic research collaborators upon reasonable request after a review process by the research team to ensure that uses of the data fall under the remit of the intended purposes set out in the privacy information and to prevent duplication of analyses. The data are not publicly available because of ethical and information governance restrictions. The full list of questions as well as other details are available on a project-specific *OxWell* Open Science Framework website along with the study protocol [73]. Full data dictionaries can be made available upon approval for access to data extracts.
