# Supplementary material for: Time Spent Gaming, Device Type, Addiction Scores, and Well-being of Adolescent English Gamers in the 2021 OxWell Survey: Latent Profile Analysis
Source: JMIR Pediatr Parent. 2022 Nov 18;5(4):e41480. doi: 10.2196/41480 (PMC9719061; doi:10.2196/41480)
Supplement: Multimedia Appendix 1 [file pediatrics_v5i4e41480_app1.docx]

Supplementary Materials

**Table S1**

*Sample attrition characteristics per predictor variable*

|  | Eligible OxWell sample (*N=*20780) | Missing gaming data (*N=*8055) | Nongamers (*N=*8755) | Gamers (*N=*3970) |
| --- | --- | --- | --- | --- |
| Well-being (WEMWBS)** | | | | |
| *M* (*SD*) | 45.1 (11.0) | 46.4 (10.9) | 44.5 (10.7) | 43.8 (11.6) |
| Missing | 1381 (6.6%) | 870 (10.8%) | 344 (3.9%) | 167 (4.2%) |
| Age** | | | | |
| 12-16 years old | 19337 (93.1%) | 7724 (95.9%) | 7849 (89.7%) | 3764 (94.8%) |
| 17-18 years old | 1443 (6.9%) | 331 (4.1%) | 906 (10.3%) | 206 (5.2%) |
| Gender** | | | | |
| Female | 10979 (52.8%) | 3991 (49.5%) | 5551 (63.4%) | 1437 (36.2%) |
| Male | 8659 (41.7%) | 3667 (45.5%) | 2746 (31.4%) | 2246 (56.6%) |
| Missing/Other | 1142 (5.5%) | 397 (4.9%) | 458 (5.2%) | 287 (7.2%) |
| Late gaming** | | | | |
| Rarely | 7454 (35.9%) | 2663 (33.1%) | 4401 (50.3%) | 390 (9.8%) |
| At least sometimes | 12468 (60.0%) | 4938 (61.3%) | 4032 (46.1%) | 3498 (88.1%) |
| Missing | 858 (4.1%) | 454 (5.6%) | 322 (3.7%) | 82 (2.1%) |
| Tried online gambling** | | | | |
| No | 11605 (55.8%) | 407 (5.1%) | 7959 (90.9%) | 3239 (81.6%) |
| Yes | 792 (3.8%) | 41 (0.5%) | 326 (3.7%) | 425 (10.7%) |
| Missing | 8383 (40.3%) | 7607 (94.4%) | 470 (5.4%) | 306 (7.7%) |
| In-game purchases** | | | | |
| No | 4944 (23.8%) | 88 (1.1%) | 4138 (47.3%) | 718 (18.1%) |
| Yes | 7849 (37.8%) | 230 (2.9%) | 4496 (51.4%) | 3123 (78.7%) |
| Missing | 7987 (38.4%) | 7737 (96.1%) | 121 (1.4%) | 129 (3.2%) |
| School community** | | | | |
| No | 12912 (62.1%) | 3809 (47.3%) | 6063 (69.3%) | 3040 (76.6%) |
| Yes | 4507 (21.7%) | 1694 (21.0%) | 2139 (24.4%) | 674 (17.0%) |
| Missing | 3361 (16.2%) | 2552 (31.7%) | 553 (6.3%) | 256 (6.4%) |
| Friendships* | | | | |
| Easy | 10238 (49.3%) | 3723 (46.2%) | 4551 (52.0%) | 1964 (49.5%) |
| Difficult | 8385 (40.4%) | 2791 (34.6%) | 3812 (43.5%) | 1782 (44.9%) |
| Missing | 2157 (10.4%) | 1541 (19.1%) | 392 (4.5%) | 224 (5.6%) |
| Detention** | | | | |
| Once or twice | 11693 (56.3%) | 848 (10.5%) | 7733 (88.3%) | 3112 (78.4%) |
| At least several times | 1888 (9.1%) | 219 (2.7%) | 902 (10.3%) | 767 (19.3%) |
| Missing | 7199 (34.6%) | 6988 (86.8%) | 120 (1.4%) | 91 (2.3%) |
| Aggression** | | | | |
| No | 15926 (76.6%) | 4724 (58.6%) | 7886 (90.1%) | 3316 (83.5%) |
| Yes | 1761 (8.5%) | 584 (7.3%) | 660 (7.5%) | 517 (13.0%) |
| Missing | 3093 (14.9%) | 2747 (34.1%) | 209 (2.4%) | 137 (3.5%) |
| Bullying** | | | | |
| Not bullied | 18319 (88.2%) | 6496 (80.6%) | 8204 (93.7%) | 3619 (91.2%) |
| Bullied | 1432 (6.9%) | 639 (7.9%) | 490 (5.6%) | 303 (7.6%) |
| Missing | 1029 (5.0%) | 920 (11.4%) | 61 (0.7%) | 48 (1.2%) |
| Exercise** | | | | |
| 1 hour or less | 2176 (10.5%) | 861 (10.7%) | 814 (9.3%) | 501 (12.6%) |
| > 1 hour | 17305 (83.3%) | 6510 (80.8%) | 7525 (86.0%) | 3270 (82.4%) |
| Missing | 1299 (6.3%) | 684 (8.5%) | 416 (4.8%) | 199 (5.0%) |
| Sense of safety** | | | | |
| Safe | 15330 (73.8%) | 4159 (51.6%) | 7749 (88.5%) | 3422 (86.2%) |
| Unsafe | 1759 (8.5%) | 389 (4.8%) | 899 (10.3%) | 471 (11.9%) |
| Missing | 3691 (17.8%) | 3507 (43.5%) | 107 (1.2%) | 77 (1.9%) |
| Food poverty** | | | | |
| No | 17436 (83.9%) | 6620 (82.2%) | 7582 (86.6%) | 3234 (81.5%) |
| Yes | 3061 (14.7%) | 1298 (16.1%) | 1080 (12.3%) | 683 (17.2%) |
| Missing | 283 (1.4%) | 137 (1.7%) | 93 (1.1%) | 53 (1.3%) |
| Abuse** | | | | |
| No | 17936 (86.3%) | 7916 (98.3%) | 6979 (79.7%) | 3041 (76.6%) |
| Yes | 2844 (13.7%) | 139 (1.7%) | 1776 (20.3%) | 929 (23.4%) |
| Anxiety** | | | | |
| Below threshold | 12753 (61.4%) | 2515 (31.2%) | 7161 (81.8%) | 3007 (77.5%) |
| Above threshold | 2011 (9.7%) | 384 (4.8%) | 1066 (12.2%) | 561 (14.1%) |
| Missing | 6016 (29.0%) | 5156 (64.0%) | 528 (6.0%) | 332 (8.4%) |
| Depression** | | | | |
| Below threshold | 12359 (59.5%) | 2486 (30.9%) | 6974 (79.7%) | 2899 (73.0%) |
| Above threshold | 2428 (11.7%) | 433 (5.4%) | 1252 (14.3%) | 743 (18.7%) |
| Missing | 5993 (28.8%) | 5136 (63.8%) | 529 (6.0%) | 328 (8.3%) |
| Insomnia** | | | | |
| No | 18032 (86.8%) | 6985 (86.7%) | 7701 (88.0%) | 3346 (84.3%) |
| Yes | 2291 (11.0%) | 784 (9.7%) | 946 (10.8%) | 561 (14.1%) |
| Missing | 457 (2.2%) | 286 (3.6%) | 108 (1.2%) | 63 (1.6%) |
| Loneliness | | | | |
| Not lonely | 11299 (54.4%) | 4185 (52.0%) | 4942 (56.4%) | 2172 (54.7%) |
| Lonely | 8012 (38.6%) | 2603 (32.3%) | 3689 (42.1%) | 1720 (43.3%) |
| Missing | 1469 (7.1%) | 1267 (15.7%) | 124 (1.4%) | 78 (2.0%) |
| Self-harm* | | | | |
| No | 8147 (39.2%) | 229 (2.8%) | 5570 (63.6%) | 2348 (59.1%) |
| Yes | 2352 (11.3%) | 71 (0.9%) | 1543 (17.6%) | 738 (18.6%) |
| Missing | 10281 (49.5%) | 7755 (96.3%) | 1642 (18.8%) | 884 (22.3%) |

*Note***.** In total, 33,453 students accessed the OxWell survey in 2021. Of this group, 3,240 were removed due to spending less than 10 min on the survey as questions of interest could not be reasonably reached in that time, 276 were removed due to not providing age information, and 9,157 were removed due to falling outside the 12-18 age range as the gaming questions were only presented to students in school years 8-13. This has resulted in a sample of 20,780 eligible participants based on pre-defined inclusion criteria (https://osf.io/s7apd). ***p* < .001, **p* < .05 for χ^2^ comparison between ‘gamers’ and ‘Nongamers’.

**Table S2**

*OxWell 2021 measures included as predictor variables in the current study and their associated coding*

| Predictor variable | Question | Response coding | In-text categories |
| --- | --- | --- | --- |
| Age | *“How old are you?”* | 0 = 12-16 | 12-16 years old |
|  |  | 1 = 17-18 | 17-18 years old |
| Gender | *“Are you male or female?”* | 0 = Female | Female |
|  |  | 1 = Male | Male |
|  |  | NA = Other/ Prefer not to answer/ Missing | Missing/Other |
| Late gaming | *“How often do you play video games in the hour before you intend to go to sleep (including games on consoles, computer, tablet, mobile phone or other portable gaming device)?”* | 0 = Never/ Rarely (1-2 times a month) | Rarely |
|  |  | 1= Sometimes (1-2 times a week)/ Often (3-4 times a week)/ Daily | At least sometimes |
|  |  | NA = Missing | Missing |
| Tried online gambling | *“Have you ever tried online gambling?”* | 0 = No | No |
|  |  | 1 = Yes | Yes |
|  |  | NA = Missing | Missing |
| In-game purchases | *“Have you ever spent money on in-game purchases?”* | 0 = No | No |
|  |  | 1 = Yes | Yes |
|  |  | NA = Missing | Missing |
| School community | *“How much do you agree with the following statement: I identify with my school community”* | 0 = Fully Disagree/ Disagree/ Neither Agree nor disagree | No |
|  |  | 1 = Agree/ Fully Agree | Yes |
|  |  | NA = Missing | Missing |
| Friendships | *“How easy do you find it to make and keep friends?”* | 0 = Quite easy/ Very easy | Easy |
|  |  | 1 = Very difficult/ Quite difficult/ Sometimes difficult | Difficult |
|  |  | NA = Missing | Missing |
| Detention | *“During this academic year, have you ever been given a detention?”* | 0 = No/Once or twice this year, | Once or twice |
|  |  | 1 = Several times this year/ Frequently this year | Several times |
|  |  | NA = Missing | Missing |
| Aggression | *“I am often aggressive or violent”* | 0 = Strongly Disagree/ Disagree/ Neither Agree nor disagree | No |
|  |  | 1 = Agree/ Strongly Agree; | Yes |
|  |  | NA = Missing | Missing |
| Bullying | *“Have you been bullied in the last year?”* | 0 = No/ Not often (e.g. once or twice) | Not bullied |
|  |  | 1 = Sometimes (e.g. monthly)/ Quite often (e.g. weekly)/ Most days | Bullied |
|  |  | NA = Missing | Missing |
| Exercise | *“About how many hours physical activity or exercise do you do over a whole week (in and out of school, during term-time)?”* | 0 = 1 hour or less | 1 hour or less |
|  |  | 1 = 3 hours/ 6 hours/ 9 hours/ 12 hours or more | > 1 hour |
|  |  | Missing = Missing | Missing |
| Sense of safety | *“How safe do you feel at home or the place where you live?”* | 0 = Safe/ Very safe | Safe |
|  |  | 1 = Very unsafe/ Unsafe/ Neither safe nor unsafe | Unsafe |
|  |  | NA = Missing | Missing |
| Food poverty | *“Some young people go to school or to bed hungry because there is not enough food at home. How often does this happen to you?”* | 0 = Not at all | No |
|  |  | 1 = Once or twice/ Sometimes/ Most days/ Every day; | Yes |
|  |  | NA = Missing | Missing |
| Abuse | - *“Did a parent or other adult in the household hit, beat, kick or physically try to hurt you in any way?”* - *“Did a parent or other adult in the household swear at you, insult you, humiliate you, threaten you or make you feel unwanted?”* - *“Did someone at least five years older than you or an adult touch or fondle you or have you touch their body in a sexual way?”* - *“Did your parent/caregiver for long periods of time not provide you with enough food or drink, clean clothes, or a clean and warm place to live?”* - *“Were there times when there was no adult living with you who made you feel loved?”*   - *“Did you see or hear one of your parents/carers being slapped, kicked, punched, beaten or deliberately hurt by a partner or ex?”* | 1 = Ticked “Yes” box for any of the items | Yes |
|  |  | 0 = Not ticked any of the “Yes” boxes | No |
|  |  |  |  |
|  |  |  |  |
|  |  |  |  |
|  |  |  |  |
| Anxiety | *RCADS-25* (Ebesutani et al., 2012)*: anxiety sub-scale* | 0 = <70 T-score | Below threshold |
|  |  | 1 = ≥70 T-score | Above threshold |
|  |  | NA = Missing | Missing |
| Depression | *RCADS-25* (Ebesutani et al., 2012)*: depression sub-scale* | 0 = <70 T-score | Below threshold |
|  |  | 1 = ≥70 T-score | Above threshold |
|  |  | NA = Missing | Missing |
| Insomnia | *SCI-02* (Luik et al., 2019) | 0 = 3-8 sum score | No |
|  |  | 1 = 0-2 sum score | Yes |
|  |  | NA = Missing | Missing |
| Loneliness | *UCLA-SF3* (Hughes et al., 2004; Russell, 1996) | 0 = 3-5 sum score | Not Lonely |
|  |  | 1 = 6-9 sum score | Lonely |
|  |  | NA = Missing | Missing |
| Self-harm | *“Have you ever deliberately self-harmed (for example by taking an overdose or deliberately injuring yourself in some other way)?”* | 0 = No | No |
|  |  | 1 = Yes | Yes |
|  |  | NA = Prefer not to say/ Not sure what this means/Missing | Missing |

**Table S3**

*Classification variable comparison for participants included in and excluded from the prediction sub-sample*

|  | Excluded (*N=*1804) | Included (*N=*2172) | *t* | *df* | *p* |
| --- | --- | --- | --- | --- | --- |
| Computer gaming |  |  |  |  |  |
| *M* (*SD*) | 2.76 (1.49) | 2.93 (1.40) | -3.64 | 3599.90 | <.001 |
| Missing | 74 (4.1%) | 19 (0.9%) |  |  |  |
| Phone gaming |  |  |  |  |  |
| *M* (*SD*) | 1.67 (1.22) | 1.52 (1.20) | 3.66 | 3605.70 | <.001 |
| Missing | 109 (6.0%) | 60 (2.8%) |  |  |  |
| Gaming addiction (GAS) |  |  |  |  |  |
| *M* (*SD*) | 2.63 (0.980) | 2.47 (0.908) | 5.02 | 3273.00 | <.001 |
| Missing | 214 (11.9%) | 37 (1.7%) |  |  |  |
| Well-being (WEMWBS) |  |  |  |  |  |
| *M* (*SD*) | 41.7 (11.8) | 45.4 (11.1) | -10.01 | 3498.50 | <.001 |
| Missing | 128 (7.1%) | 45 (2.1%) |  |  |  |

**Table S4**

*Predictor variable comparison for participants included in and excluded from the prediction sub-sample*

|  | Excluded (*N=*1804) | Included (*N=*2172) | χ^2^(1) | *p* |
| --- | --- | --- | --- | --- |
| Age |  | | 9.08 | .003 |
| 12-16 years old | 1732 (96.0%) | 2038 (93.8%) |  |  |
| 17-18 years old | 72 (4.0%) | 134 (6.2%) |  |  |
| Gender | | | 38.76 | <.001 |
| Female | 683 (37.9%) | 756 (34.8%) |  |  |
| Male | 834 (46.2%) | 1416 (65.2%) |  |  |
| Missing | 287 (15.9%) | 0 (0%) |  |  |
| Late gaming |  |  | 0.42 | .517 |
| Rarely | 179 (9.9%) | 211 (9.7%) |  |  |
| At least sometimes | 1543 (85.5%) | 1961 (90.3%) |  |  |
| Missing | 82 (4.5%) | 0 (0%) |  |  |
| Tried online gambling | | | 3.19 | .074 |
| No | 1307 (72.5%) | 1938 (89.2%) |  |  |
| Yes | 191 (10.6%) | 234 (10.8%) |  |  |
| Missing | 306 (17.0%) | 0 (0%) |  |  |
| In-game purchases |  |  | 5.15 | .023 |
| No | 340 (18.8%) | 378 (17.4%) |  |  |
| Yes | 1332 (73.8%) | 1794 (82.6%) |  |  |
| Missing | 132 (7.3%) | 0 (0%) |  |  |
| School community | | | 27.23 | <.001 |
| No | 1325 (73.4%) | 1716 (79.0%) |  |  |
| Yes | 220 (12.2%) | 456 (21.0%) |  |  |
| Missing | 259 (14.4%) | 0 (0%) |  |  |
| Friendships |  |  | 27.53 | <.001 |
| Easy | 748 (41.5%) | 1219 (56.1%) |  |  |
| Difficult | 830 (46.0%) | 953 (43.9%) |  |  |
| Missing | 226 (12.5%) | 0 (0%) |  |  |
| Detention |  |  | 14.34 | <.001 |
| Once or twice | 1325 (73.4%) | 1790 (82.4%) |  |  |
| At least several times | 385 (21.3%) | 382 (17.6%) |  |  |
| Missing | 94 (5.2%) | 0 (0%) |  |  |
| Aggression |  |  | 29.98 | <.001 |
| No | 1380 (76.5%) | 1937 (89.2%) |  |  |
| Yes | 282 (15.6%) | 235 (10.8%) |  |  |
| Missing | 142 (7.9%) | 0 (0%) |  |  |
| Bullying |  |  | 15.37 | <.001 |
| Not bullied | 1586 (87.9%) | 2037 (93.8%) |  |  |
| Bullied | 169 (9.4%) | 135 (6.2%) |  |  |
| Missing | 49 (2.7%) | 0 (0%) |  |  |
| Exercise |  |  | 11.74 | .001 |
| 1 hour or less | 249 (13.8%) | 253 (11.6%) |  |  |
| > 1 hour | 1354 (75.1%) | 1919 (88.4%) |  |  |
| Missing | 201 (11.1%) | 0 (0%) |  |  |
| Sense of safety |  |  | 35.26 | <.001 |
| Safe | 1456 (80.7%) | 1970 (90.7%) |  |  |
| Unsafe | 269 (14.9%) | 202 (9.3%) |  |  |
| Missing | 79 (4.4%) | 0 (0%) |  |  |
| Food poverty | | | 21.50 | <.001 |
| No | 1390 (77.1%) | 1849 (85.1%) |  |  |
| Yes | 360 (20.0%) | 323 (14.9%) |  |  |
| Missing | 54 (3.0%) | 0 (0%) |  |  |
| Abuse | | | 14.16 | <.001 |
| No | 1332 (73.8%) | 1715 (79.0%) |  |  |
| Yes | 472 (26.2%) | 457 (21.0%) |  |  |
| Anxiety |  |  | 10.22 | .001 |
| Below threshold | 1205 (66.8%) | 1873 (86.2%) |  |  |
| Above threshold | 264 (14.6%) | 299 (13.8%) |  |  |
| Missing | 335 (18.6%) | 0 (0%) |  |  |
| Depression |  |  | 11.54 | <.001 |
| Below threshold | 1113 (61.7%) | 1789 (82.4%) |  |  |
| Above threshold | 361 (20.0%) | 383 (17.6%) |  |  |
| Missing | 330 (18.3%) | 0 (0%) |  |  |
| Insomnia |  |  | 25.00 | <.001 |
| No | 1432 (79.4%) | 1919 (88.4%) |  |  |
| Yes | 308 (17.1%) | 253 (11.6%) |  |  |
| Missing | 64 (3.5%) | 0 (0%) |  |  |
| Loneliness |  |  | 66.45 | <.001 |
| Not lonely | 836 (46.3%) | 1338 (61.6%) |  |  |
| Lonely | 888 (49.2%) | 834 (38.4%) |  |  |
| Missing | 80 (4.4%) | 0 (0%) |  |  |
| Self-harm |  |  | 37.97 | <.001 |
| No | 632 (35.0%) | 1720 (79.2%) |  |  |
| Yes | 287 (15.9%) | 452 (20.8%) |  |  |
| Missing | 885 (49.1%) | 0 (0%) |  |  |

**Table S5**

*Model fit statistics and diagnostic criteria for models with 2 to 6 classes*

|  | *df* | BIC | LL | VLMR- LRT | | Entropy | Smallest class |
| --- | --- | --- | --- | --- | --- | --- | --- |
|  |  |  |  | -2LL | *P* |  |  |
| 2 Class | 19 | 61989 | -30915.88 | 2542.33 | <.001 | .919 | 21% |
| 3 Class | 24 | 60991 | -30395.98 | 1039.82 | <.001 | .955 | 21% |
| 4 Class | 29 | 58587 | -29173.36 | 2445.24 | <.001 | .981 | 14% |
| 5 Class | 34 | 58582 | -29149.90 | 46.91 | .22 | .907 | 4% |
| **6 Class** | **39** | **58509** | **-29092.86** | **102.93** | **<.001** | **.897** | **2%** |

*Note.* Bold text indicates model met fit criteria. BIC = Bayesian information criterion; LL = log-likelihood; -2LL = 2 times the log-likelihood difference; LRT = Vuong-Lo-Mendell- Rubin adjusted likelihood ratio test. The 5 Class model had starting values (100000/25000) and iterations (5000) increased to achieve convergence at global maxima.

**Table S6**

*Missing data on classification variables per profile*

|  | Adaptive computer gamers  (*N=*1728) | Maladaptive computer gamers (*N=*245) | Maladaptive phone gamers (*N=*69) | Unknown device gamers  (*N=*478) | Casual computer gamers  (*N=*873) | Casual phone gamers  (*N=*577) |
| --- | --- | --- | --- | --- | --- | --- |
| Computer gaming | 72 (4.2%) | 7 (2.9%) | 0 (0.0%) | 8 (1.7%) | 0 (0.0%) | 0 (0.0%) |
| Phone gaming | 81 (4.7%) | 13 (5.3%) | 0 (0.0%) | 16 (3.3%) | 25 (2.9%) | 28 (4.9%) |
| GAS | 108 (6.3%) | 2 (0.8%) | 0 (0.0%) | 60 (12.6%) | 42 (4.8%) | 33 (5.7%) |
| WEMWBS | 65 (3.8%) | 6 (2.4%) | 1 (1.4%) | 40 (8.4%) | 24 (2.7%) | 31 (5.4%) |

Table S7

Membership in each latent class according to background characteristics

|  | Adaptive computer gamers  (*N=*1728) | Maladaptive computer gamers (*N=*245) | Maladaptive phone gamers (*N=*69) | Unknown device gamers  (*N=*478) | Casual computer gamers  (*N=*873) | Casual phone gamers  (*N=*577) |
| --- | --- | --- | --- | --- | --- | --- |
| Age |  |  |  |  |  |  |
| 12-16 years old | 1643 (95.1%) | 235 (95.9%) | 65 (94.2%) | 446 (93.3%) | 828 (94.8%) | 547 (94.8%) |
| 17-18 years old | 85 (4.9%) | 10 (4.1%) | 4 (5.8%) | 32 (6.7%) | 45 (5.2%) | 30 (5.2%) |
| Gender |  |  |  |  |  |  |
| Female | 340 (19.7%) | 76 (31.0%) | 43 (62.3%) | 390 (81.6%) | 257 (29.4%) | 331 (57.4%) |
| Male | 1274 (73.7%) | 142 (58.0%) | 16 (23.2%) | 59 (12.3%) | 558 (63.9%) | 197 (34.1%) |
| Missing/Other | 114 (6.6%) | 27 (11.0%) | 10 (14.5%) | 29 (6.1%) | 58 (6.6%) | 49 (8.5%) |
| Late gaming | |  |  |  |  |  |
| Rarely | 93 (5.4%) | 8 (3.3%) | 4 (5.8%) | 129 (27.0%) | 78 (8.9%) | 78 (13.5%) |
| At least sometimes | 1604 (92.8%) | 232 (94.7%) | 64 (92.8%) | 339 (70.9%) | 777 (89.0%) | 482 (83.5%) |
| Missing | 31 (1.8%) | 5 (2.0%) | 1 (1.4%) | 10 (2.1%) | 18 (2.1%) | 17 (2.9%) |
| Tried online gambling | | |  |  |  |  |
| No | 1369 (79.2%) | 165 (67.3%) | 61 (88.4%) | 422 (88.3%) | 739 (84.7%) | 483 (83.7%) |
| Yes | 216 (12.5%) | 57 (23.3%) | 7 (10.1%) | 16 (3.3%) | 89 (10.2%) | 40 (6.9%) |
| Missing | 143 (8.3%) | 23 (9.4%) | 1 (1.4%) | 40 (8.4%) | 45 (5.2%) | 54 (9.4%) |
| In-game purchases | |  |  |  |  |  |
| No | 145 (8.4%) | 14 (5.7%) | 24 (34.8%) | 246 (51.5%) | 124 (14.2%) | 165 (28.6%) |
| Yes | 1532 (88.7%) | 226 (92.2%) | 44 (63.8%) | 196 (41.0%) | 731 (83.7%) | 394 (68.3%) |
| Missing | 51 (3.0%) | 5 (2.0%) | 1 (1.4%) | 36 (7.5%) | 18 (2.1%) | 18 (3.1%) |
| School community | | | |  |  |  |
| No | 1306 (75.6%) | 215 (87.8%) | 61 (88.4%) | 367 (76.8%) | 658 (75.4%) | 433 (75.0%) |
| Yes | 291 (16.8%) | 21 (8.6%) | 7 (10.1%) | 79 (16.5%) | 168 (19.2%) | 108 (18.7%) |
| Missing | 131 (7.6%) | 9 (3.7%) | 1 (1.4%) | 32 (6.7%) | 47 (5.4%) | 36 (6.2%) |
| Friendships | |  |  |  |  |  |
| Easy | 938 (54.3%) | 90 (36.7%) | 21 (30.4%) | 227 (47.5%) | 431 (49.4%) | 257 (44.5%) |
| Difficult | 689 (39.9%) | 140 (57.1%) | 46 (66.7%) | 215 (45.0%) | 401 (45.9%) | 291 (50.4%) |
| Missing | 101 (5.8%) | 15 (6.1%) | 2 (2.9%) | 36 (7.5%) | 41 (4.7%) | 29 (5.0%) |
| Detention |  |  |  |  |  |  |
| Once or twice | 1318 (76.3%) | 163 (66.5%) | 59 (85.5%) | 388 (81.2%) | 701 (80.3%) | 483 (83.7%) |
| At least several times | 359 (20.8%) | 76 (31.0%) | 10 (14.5%) | 84 (17.6%) | 154 (17.6%) | 84 (14.6%) |
| Missing | 51 (3.0%) | 6 (2.4%) | 0 (0%) | 6 (1.3%) | 18 (2.1%) | 10 (1.7%) |
| Aggression | |  |  |  |  |  |
| No | 1462 (84.6%) | 156 (63.7%) | 55 (79.7%) | 412 (86.2%) | 747 (85.6%) | 484 (83.9%) |
| Yes | 209 (12.1%) | 81 (33.1%) | 13 (18.8%) | 48 (10.0%) | 97 (11.1%) | 69 (12.0%) |
| Missing | 57 (3.3%) | 8 (3.3%) | 1 (1.4%) | 18 (3.8%) | 29 (3.3%) | 24 (4.2%) |
| Bullying |  |  |  |  |  |  |
| Not bullied | 1583 (91.6%) | 198 (80.8%) | 61 (88.4%) | 440 (92.1%) | 813 (93.1%) | 524 (90.8%) |
| Bullied | 124 (7.2%) | 43 (17.6%) | 7 (10.1%) | 30 (6.3%) | 54 (6.2%) | 45 (7.8%) |
| Missing | 21 (1.2%) | 4 (1.6%) | 1 (1.4%) | 8 (1.7%) | 6 (0.7%) | 8 (1.4%) |
| Exercise | |  |  |  |  |  |
| 1 hour or less | 218 (12.6%) | 44 (18.0%) | 12 (17.4%) | 76 (15.9%) | 79 (9.0%) | 72 (12.5%) |
| > 1 hour | 1413 (81.8%) | 185 (75.5%) | 56 (81.2%) | 380 (79.5%) | 757 (86.7%) | 479 (83.0%) |
| Missing | 97 (5.6%) | 16 (6.5%) | 1 (1.4%) | 22 (4.6%) | 37 (4.2%) | 26 (4.5%) |
| Sense of safety | |  |  |  |  |  |
| Safe | 1503 (87.0%) | 174 (71.0%) | 54 (78.3%) | 426 (89.1%) | 791 (90.6%) | 474 (82.1%) |
| Unsafe | 186 (10.8%) | 65 (26.5%) | 13 (18.8%) | 44 (9.2%) | 70 (8.0%) | 93 (16.1%) |
| Missing | 39 (2.3%) | 6 (2.4%) | 2 (2.9%) | 8 (1.7%) | 12 (1.4%) | 10 (1.7%) |
| Food poverty | | |  |  |  |  |
| No | 1435 (83.0%) | 153 (62.4%) | 51 (73.9%) | 425 (88.9%) | 702 (80.4%) | 468 (81.1%) |
| Yes | 269 (15.6%) | 86 (35.1%) | 17 (24.6%) | 47 (9.8%) | 164 (18.8%) | 100 (17.3%) |
| Missing | 24 (1.4%) | 6 (2.4%) | 1 (1.4%) | 6 (1.3%) | 7 (0.8%) | 9 (1.6%) |
| Abuse | | | |  |  |  |
| No | 1402 (81.1%) | 145 (59.2%) | 34 (49.3%) | 357 (74.7%) | 692 (79.3%) | 411 (71.2%) |
| Yes | 326 (18.9%) | 100 (40.8%) | 35 (50.7%) | 121 (25.3%) | 181 (20.7%) | 166 (28.8%) |
| Anxiety |  |  |  |  |  |  |
| Below threshold | 1414 (81.8%) | 133 (54.3%) | 39 (56.5%) | 357 (74.7%) | 704 (80.6%) | 430 (74.5%) |
| Above threshold | 180 (10.4%) | 84 (34.3%) | 19 (27.5%) | 85 (17.8%) | 101 (11.6%) | 92 (15.9%) |
| Missing | 134 (7.8%) | 28 (11.4%) | 11 (15.9%) | 36 (7.5%) | 68 (7.8%) | 55 (9.5%) |
| Depression | |  |  |  |  |  |
| Below threshold | 1346 (77.9%) | 120 (49.0%) | 36 (52.2%) | 333 (69.7%) | 668 (76.5%) | 396 (68.6%) |
| Above threshold | 248 (14.4%) | 97 (39.6%) | 23 (33.3%) | 110 (23.0%) | 139 (15.9%) | 126 (21.8%) |
| Missing | 134 (7.8%) | 28 (11.4%) | 10 (14.5%) | 35 (7.3%) | 66 (7.6%) | 55 (9.5%) |
| Insomnia |  |  |  |  |  |  |
| No | 1490 (86.2%) | 166 (67.8%) | 47 (68.1%) | 402 (84.1%) | 755 (86.5%) | 486 (84.2%) |
| Yes | 200 (11.6%) | 76 (31.0%) | 21 (30.4%) | 72 (15.1%) | 108 (12.4%) | 84 (14.6%) |
| Missing | 38 (2.2%) | 3 (1.2%) | 1 (1.4%) | 4 (0.8%) | 10 (1.1%) | 7 (1.2%) |
| Loneliness | |  |  |  |  |  |
| Not lonely | 1061 (61.4%) | 80 (32.7%) | 22 (31.9%) | 233 (48.7%) | 497 (56.9%) | 279 (48.4%) |
| Lonely | 625 (36.2%) | 161 (65.7%) | 47 (68.1%) | 233 (48.7%) | 364 (41.7%) | 290 (50.3%) |
| Missing | 42 (2.4%) | 4 (1.6%) | 0 (0%) | 12 (2.5%) | 12 (1.4%) | 8 (1.4%) |
| Self-harm |  |  |  |  |  |  |
| No | 1128 (65.3%) | 99 (40.4%) | 23 (33.3%) | 245 (51.3%) | 543 (62.2%) | 310 (53.7%) |
| Yes | 268 (15.5%) | 80 (32.7%) | 26 (37.7%) | 109 (22.8%) | 140 (16.0%) | 115 (19.9%) |
| Missing | 332 (19.2%) | 66 (26.9%) | 20 (29.0%) | 124 (25.9%) | 190 (21.8%) | 152 (26.3%) |

Table S8

Relative odds ratios and 95% confidence intervals from multinomial logistic regression comparing characteristics of latent class membership

|  |  | Maladaptive  computer  gamers |  | Maladaptive  phone  gamers |  | Unknown  device  gamers |  | Casual  Compute  r gamers |  | Casual  phone  gamers |
| --- | --- | --- | --- | --- | --- | --- | --- | --- | --- | --- |
| Age: 17-18 yo (vs. 12-16 yo) | | | | | | | | | | |
|  | OR | 1.24 |  | 1.69 |  | 1.13 |  | 1.27 |  | 1.04 |
|  | CI 95% | 0.50:3.06 |  | 0.46:6.21 |  | 0.55:2.30 |  | 0.80:2.02 |  | 0.56:1.91 |
| Gender: Male (vs. Female) | | | | | | | | | | |
|  | OR | **0.51** |  | **0.08** |  | **0.04** |  | **0.50** |  | **0.14** |
|  | CI 95% | **0.30:0.88** |  | **0.03:0.21** |  | **0.03:0.06** |  | **0.38:0.67** |  | **0.10:0.20** |
| Late gaming: At least sometimes (vs. Rarely) | | | | | | | | | | |
|  | OR | 1.44 |  | 0.45 |  | **0.14** |  | **0.45** |  | **0.31** |
|  | CI 95% | 0.39:5.30 |  | 0.11:1.88 |  | **0.09:0.24** |  | **0.30:0.67** |  | **0.19:0.50** |
| Tried online gambling: Yes (vs. No) | | | | |  |  |  |  |  |  |
|  | OR | **2.18** |  | 0.48 |  | 0.66 |  | 0.88 |  | 0.92 |
|  | CI 95% | **1.24:3.81** |  | 0.07:3.45 |  | 0.30:1.47 |  | 0.62:1.26 |  | 0.56:1.51 |
| In-game purchases: Yes (vs. No) | | | | | | | | | | |
|  | OR | 1.19 |  | **0.40** |  | **0.14** |  | **0.60** |  | **0.31** |
|  | CI 95% | 0.51:2.73 |  | **0.17:0.95** |  | **0.09:0.21** |  | **0.42:0.86** |  | **0.21:0.45** |
| School community: Yes (vs. No) | | | | | |  |  |  |  |  |
|  | OR | 0.75 |  | 0.58 |  | 1.45 |  | 1.20 |  | **1.52** |
|  | CI 95% | 0.36:1.58 |  | 0.14:2.41 |  | 0.93:2.24 |  | 0.91:1.57 |  | **1.07:2.15** |
| Friendships: Difficult (vs. Easy) | | | | | | | | | | |
|  | OR | 1.45 |  | 1.36 |  | 0.86 |  | **1.39** |  | 1.23 |
|  | CI 95% | 0.88:2.40 |  | 0.59:3.13 |  | 0.59:1.26 |  | **1.09:1.76** |  | 0.91:1.68 |
| Detention: At least several times (vs. Once or twice) | | | | | | |  |  |  |  |
|  | OR | 1.25 |  | 1.04 |  | 1.47 |  | 0.88 |  | 1.07 |
|  | CI 95% | 0.73:2.13 |  | 0.37:2.91 |  | 0.91:2.37 |  | 0.65:1.19 |  | 0.72:1.57 |
| Aggression: Yes (vs. No) | | | |  |  |  |  |  |  |  |
|  | OR | **2.83** |  | 1.32 |  | 1.48 |  | 1.03 |  | 1.03 |
|  | CI 95% | **1.65:4.88** |  | 0.44:4.00 |  | 0.83:2.66 |  | 0.70:1.53 |  | 0.63:1.67 |
| Bullying: Bullied (vs. Not bullied) | | | | |  |  |  |  |  |  |
|  | OR | 0.56 |  | 0.97 |  | 0.85 |  | 0.65 |  | 0.79 |
|  | CI 95% | 0.25:1.25 |  | 0.27:3.50 |  | 0.40:1.81 |  | 0.39:1.06 |  | 0.44:1.41 |
| Exercise: > 1 hour (vs. 1 hour or less) | | | | | | |  |  |  |  |
|  | OR | 1.10 |  | 0.64 |  | 0.86 |  | **1.63** |  | 1.57 |
|  | CI 95% | 0.58:2.09 |  | 0.25:1.61 |  | 0.52:1.43 |  | **1.12:2.37** |  | 1.00:2.49 |
| Sense of safety: Unsafe (vs. Safe) | | | | | | | | | | |
|  | OR | 1.50 |  | 0.55 |  | **0.30** |  | **0.53** |  | 1.16 |
|  | CI 95% | 0.81:2.78 |  | 0.18:1.69 |  | **0.14:0.62** |  | **0.33:0.85** |  | 0.72:1.89 |
| Food poverty: Yes (vs. No) | | | | |  |  |  |  |  |  |
|  | OR | 1.37 |  | 1.50 |  | 0.64 |  | 1.24 |  | 1.20 |
|  | CI 95% | 0.80:2.35 |  | 0.62:3.67 |  | 0.36:1.14 |  | 0.90:1.70 |  | 0.80:1.77 |
| Abuse: Yes (vs. No) | | | | | |  |  |  |  |  |
|  | OR | 1.06 |  | **3.18** |  | 1.26 |  | 1.13 |  | 1.31 |
|  | CI 95% | 0.61:1.84 |  | **1.34:7.55** |  | 0.79:2.01 |  | 0.83:1.55 |  | 0.90:1.91 |
| Anxiety: Above (vs. Below) threshold | | | | |  |  |  |  |  |  |
|  | OR | **2.25** |  | 0.91 |  | 1.51 |  | 1.18 |  | 0.94 |
|  | CI 95% | **1.23:4.12** |  | 0.33:2.54 |  | 0.85:2.66 |  | 0.78:1.79 |  | 0.58:1.53 |
| Depression: Above (vs. Below) threshold | | | | | |  |  |  |  |  |
|  | OR | 1.30 |  | 0.69 |  | 0.86 |  | 0.82 |  | 0.88 |
|  | CI 95% | 0.68:2.50 |  | 0.24:1.98 |  | 0.48:1.57 |  | 0.54:1.24 |  | 0.54:1.43 |
| Insomnia: Yes (vs. No) | | |  |  |  |  |  |  |  |  |
|  | OR | 1.12 |  | 1.16 |  | 0.62 |  | 0.92 |  | 0.64 |
|  | CI 95% | 0.61:2.06 |  | 0.43:3.10 |  | 0.34:1.14 |  | 0.61:1.39 |  | 0.39:1.05 |
| Loneliness: Lonely (vs. Not Lonely) | | | | |  |  |  |  |  |  |
|  | OR | 1.55 |  | 1.48 |  | 1.19 |  | 1.24 |  | 1.38 |
|  | CI 95% | 0.87:2.76 |  | 0.59:3.68 |  | 0.77:1.82 |  | 0.94:1.63 |  | 0.98:1.95 |
| Self-harm: Yes (vs. No) | | | | | | | | | | |
|  | OR | 1.06 |  | 1.18 |  | 0.71 |  | 0.81 |  | 0.84 |
|  | CI 95% | 0.59:1.91 |  | 0.46:3.01 |  | 0.43:1.18 |  | 0.57:1.15 |  | 0.56:1.28 |

*Note*. Reference group - ‘Adaptive computer gamers’.

**Supplementary Analysis: Gamer Profiles in Comparison with Nongamers**

In order to contextualise our main findings, we have conducted an additional analysis comparing the LPA devised gamer profiles to the ‘non-gamer’ participants, who reported that they played video games for less than 3.5 hours a day and, thus, could not be included in the main analysis due to missing data on gaming behaviours. We first investigated whether participants’ well-being scores differed between the LPA defined gamer profiles and the ‘non-gamer’ group using Bonferroni corrected paired t-tests. We then carried out a multinomial logistic similarly to the analysis described in the Data Analysis section. However, due to the comparison to pre-, rather than LPA, defined group, the posterior probability weights were not included in this analysis. In other words, the analysis described here is unable to account for the potential uncertainty of the classification system.

Supplementary analysis showed that there were significant differences in well-being scores between ‘Nongamers’ and most of the gamer profiles (Figure S1 and Table 4). On average, ‘Nongamers’ reported similar well-being to that of ‘casual computer gamers’. It was statistically significantly lower than well-being reported by ‘adaptive computer gamers’, but higher than well-being of other gamer groups.

Figure S1

Means and standard deviations of well-being scores for gamer and ‘non-gamer’ groups


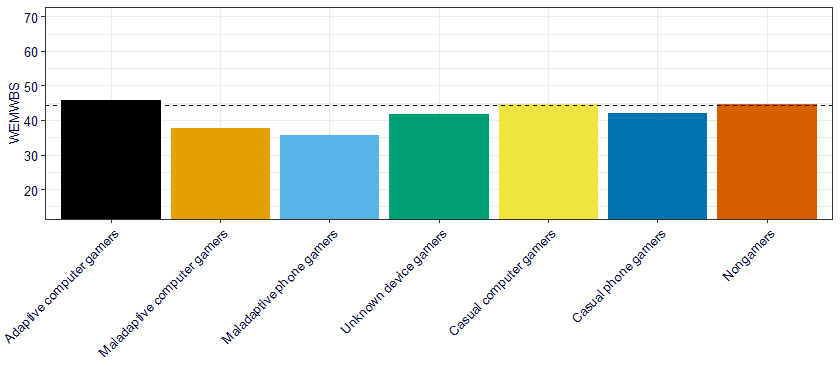


Multinomial logistic regression revealed further differences between ‘Nongamers’ and LPA defined gamer profiles (Figure S2 and Table S9). Specifically, ‘adaptive computer gamers’ were more likely than ‘Nongamers’ to be male, report engaging in late gaming before bed, trying online gambling, spending money on in-game purchases, experience depression and insomnia, but less likely to be older, identify with their school community, and exercise more than 1 hour a week. ‘Maladaptive computer gamers’ were also more likely than ‘Nongamers’ to be male, report engaging in late gaming before bed, trying online gambling, spending money on in-game purchases, experience depression or insomnia and less likely to identify with their school community. Yet, in contrast to the former group, ‘maladaptive computer gamers’ were more likely than ‘Nongamers’ to report aggressive behaviours and anxiety. In line with the findings of the main analysis, ‘maladaptive phone gamers’ were more likely than ‘Nongamers’ to report playing video games late before bed and having experienced previous abuse. ‘Casual computer gamers’ reported similar well-being to ‘Nongamers’ but were more likely to be in the 12-16-year-old age group, be male, report engaging in late gaming and spend money on in-game purchases, have difficulty making and keeping friends, feel safe where they live, and have insomnia. ‘Unknown device gamers’ were the only group that was more likely than ‘Nongamers’ to be female, they were also more likely to engage in late gaming, have experiences of detention and feel safe where they live, but less likely to report spending money on in-game purchases and exercise. Despite reporting lower well-being, ‘casual phone gamers’ were most similar to the ‘Nongamers’ in terms of their correlates with only differences occurring in their likelihood to report gaming late before sleep and spending money on in-game purchases.

Taken together with the findings from the main analysis, these findings confirm that there are qualitative differences between different groups of gamers that extend beyond effects of time spent playing video games or even gaming addiction scores. By doing so it highlights the importance of investigating differences in gaming behaviours, off-line experiences, well-being, and mental health between different types of gamers and not only in relation to those who do not game.

Figure S2

Relative odds ratios comparing likelihood of gaming profiles per hypothesized predictor variables


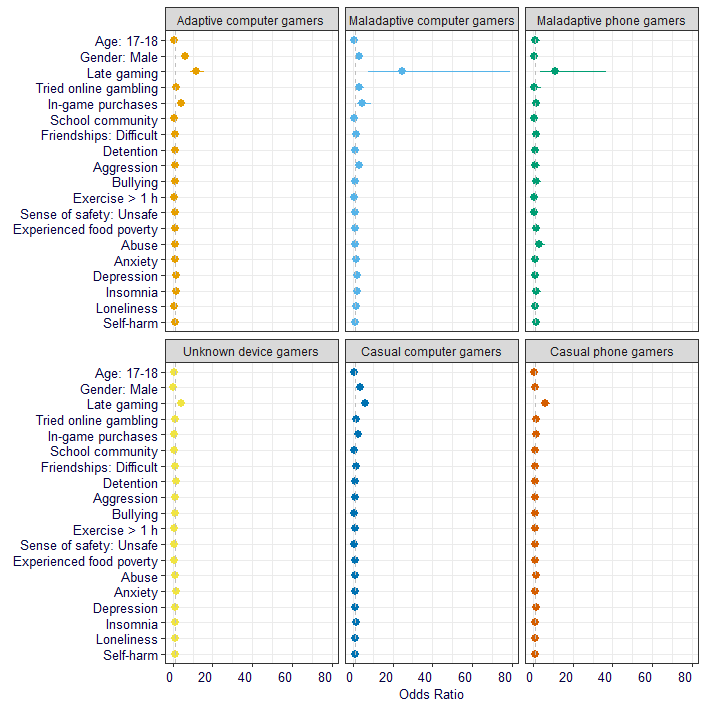


*Note*. Reference group - ‘Nongamers’. Error bars represent 95% confidence intervals.

Table S9

Relative odds ratios and 95% confidence intervals from multinomial logistic regression comparing characteristics of latent class membership to ‘Nongamers’ group

|  |  | Adaptive  computer  gamers | Maladaptive  computer  gamers | Maladaptive  phone  gamers | Unknown  device  gamers | Casual  computer  gamers | Casual  phone  gamers |
| --- | --- | --- | --- | --- | --- | --- | --- |
| Age: 17-18 (vs. 12-16) | | | |  |  |  |  |
|  | OR | **0.47** | 0.73 | 1.14 | 0.80 | **0.60** | 0.62 |
|  | CI 95% | **0.33:0.66** | 0.35:1.50 | 0.38:3.41 | 0.49:1.30 | **0.41:0.89** | 0.37:1.02 |
| Gender: Male (vs. Female) | | | |  |  |  |  |
|  | OR | **6.17** | **3.20** | 0.45 | **0.33** | **3.34** | 0.99 |
|  | CI 95% | **5.01:7.60** | **2.03:5.05** | 0.17:1.16 | **0.22:0.49** | **2.65:4.21** | 0.75:1.30 |
| Late gaming: At least sometimes (vs. Rarely) | | | | |  |  |  |
|  | OR | **11.83** | **24.69** | **11.01** | **3.91** | **6.17** | **6.15** |
|  | CI 95% | **8.78:15.94** | **7.69:79.25** | **3.30:36.78** | **2.87:5.34** | **4.60:8.29** | **4.35:8.70** |
| Tried online gambling: Yes (vs. No) | | | | |  |  |  |
|  | OR | **1.64** | **3.30** | 0.56 | 1.26 | 1.41 | 1.42 |
|  | CI 95% | **1.26:2.15** | **2.01:5.43** | 0.07:4.23 | 0.62:2.54 | 0.99:2.00 | 0.87:2.31 |
| In-game purchases: Yes (vs. No) | | | |  |  |  |  |
|  | OR | **4.34** | **4.53** | 1.55 | **0.70** | **2.81** | **1.45** |
|  | CI 95% | **3.33:5.64** | **2.24:9.18** | 0.73:3.26 | **0.53:0.93** | **2.13:3.71** | **1.10:1.91** |
| School community: Yes (vs. No) | | | | | |  |  |
|  | OR | **0.69** | **0.50** | 0.44 | 0.86 | 0.81 | 0.99 |
|  | CI 95% | **0.57:0.84** | **0.26:0.94** | 0.13:1.50 | 0.62:1.19 | 0.64:1.02 | 0.74:1.34 |
| Friendships: Difficult (vs. Easy) | | | |  |  |  |  |
|  | OR | 1.00 | 1.36 | 1.27 | 0.94 | **1.37** | 1.23 |
|  | CI 95% | 0.83:1.19 | 0.88:2.09 | 0.60:2.71 | 0.70:1.25 | **1.11:1.70** | 0.94:1.60 |
| Detention: At least several times (vs. Once or twice) | | | | | |  |  |
|  | OR | 1.15 | 1.24 | 1.15 | **1.74** | 0.96 | 1.19 |
|  | CI 95% | 0.92:1.43 | 0.78:1.99 | 0.45:2.94 | **1.19:2.54** | 0.72:1.28 | 0.84:1.70 |
| Aggression: Yes (vs. No) | | | |  |  |  |  |
|  | OR | 1.00 | **2.85** | 1.17 | 1.33 | 1.02 | 1.01 |
|  | CI 95% | 0.74:1.33 | **1.79:4.54** | 0.42:3.25 | 0.84:2.11 | 0.71:1.47 | 0.66:1.56 |
| Bullying: Bullied (vs. Not bullied) | | | | | | | |
|  | OR | 1.24 | 0.85 | 1.29 | 0.90 | 0.78 | 0.96 |
|  | CI 95% | 0.88:1.74 | 0.43:1.67 | 0.42:3.90 | 0.48:1.67 | 0.49:1.24 | 0.58:1.60 |
| Exercise: > 1 hour (vs. 1 hour or less) | | | | | | |  |
|  | OR | **0.55** | 0.58 | 0.45 | **0.54** | 0.91 | 0.91 |
|  | CI 95% | **0.42:0.71** | 0.33:1.01 | 0.20:1.02 | **0.37:0.77** | 0.64:1.30 | 0.61:1.35 |
| Sense of safety: Unsafe (vs. Safe) | | | |  |  |  |  |
|  | OR | 0.94 | 1.23 | 0.58 | **0.39** | **0.47** | 1.13 |
|  | CI 95% | 0.70:1.28 | 0.72:2.09 | 0.22:1.58 | **0.21:0.73** | **0.30:0.74** | 0.76:1.69 |
| Food poverty: Yes (vs. No) | | | | |  |  |  |
|  | OR | 0.90 | 1.20 | 1.31 | 0.67 | 1.14 | 1.10 |
|  | CI 95% | 0.71:1.15 | 0.75:1.92 | 0.58:2.99 | 0.42:1.08 | 0.85:1.52 | 0.78:1.56 |
| Abuse: Yes (vs. No) | | | | | |  |  |
|  | OR | 0.98 | 1.03 | **2.81** | 1.17 | 1.08 | 1.26 |
|  | CI 95% | 0.78:1.24 | 0.65:1.66 | **1.28:6.19** | 0.82:1.68 | 0.82:1.43 | 0.92:1.74 |
| Anxiety: Above (vs. Below) threshold | | | | |  |  |  |
|  | OR | 0.92 | **1.77** | 1.02 | 1.50 | 1.10 | 0.92 |
|  | CI 95% | 0.67:1.26 | **1.05:2.99** | 0.42:2.54 | 0.98:2.29 | 0.76:1.58 | 0.61:1.38 |
| Depression: Above (vs. Below) threshold | | | | | |  |  |
|  | OR | **1.58** | **2.00** | 1.20 | 1.31 | 1.30 | 1.41 |
|  | CI 95% | **1.15:2.19** | **1.11:3.60** | 0.46:3.18 | 0.83:2.08 | 0.88:1.90 | 0.93:2.14 |
| Insomnia: Yes (vs. No) | | | |  |  |  |  |
|  | OR | **1.70** | **1.95** | 1.64 | 0.99 | **1.53** | 1.00 |
|  | CI 95% | **1.24:2.32** | **1.14:3.31** | 0.67:4.00 | 0.61:1.59 | **1.06:2.24** | 0.65:1.54 |
| Loneliness: Lonely (vs. Not Lonely) | | | | |  |  |  |
|  | OR | 0.88 | 1.39 | 1.22 | 0.90 | 1.03 | 1.10 |
|  | CI 95% | 0.71:1.08 | 0.84:2.28 | 0.52:2.85 | 0.65:1.25 | 0.81:1.33 | 0.82:1.48 |
| Self-harm: Yes (vs. No) | | | |  |  |  |  |
|  | OR | 1.14 | 1.10 | 1.31 | 0.95 | 0.97 | 1.04 |
|  | CI 95% | 0.88:1.47 | 0.66:1.86 | 0.55:3.11 | 0.65:1.41 | 0.71:1.32 | 0.73:1.48 |

*Note*. Reference group - ‘Nongamers’. Due to the comparison to self, rather than LPA, defined group, the posterior probability weights were not included in this analysis.
